# Supplementary figures and images for: Human Cytomegalovirus Uses a Host Stress Response To Balance the Elongation of Saturated/Monounsaturated and Polyunsaturated Very-Long-Chain Fatty Acids
Source: mBio. 2021 May 4;12(3):e00167-21. doi: 10.1128/mBio.00167-21 (PMC8262922; doi:10.1128/mBio.00167-21)

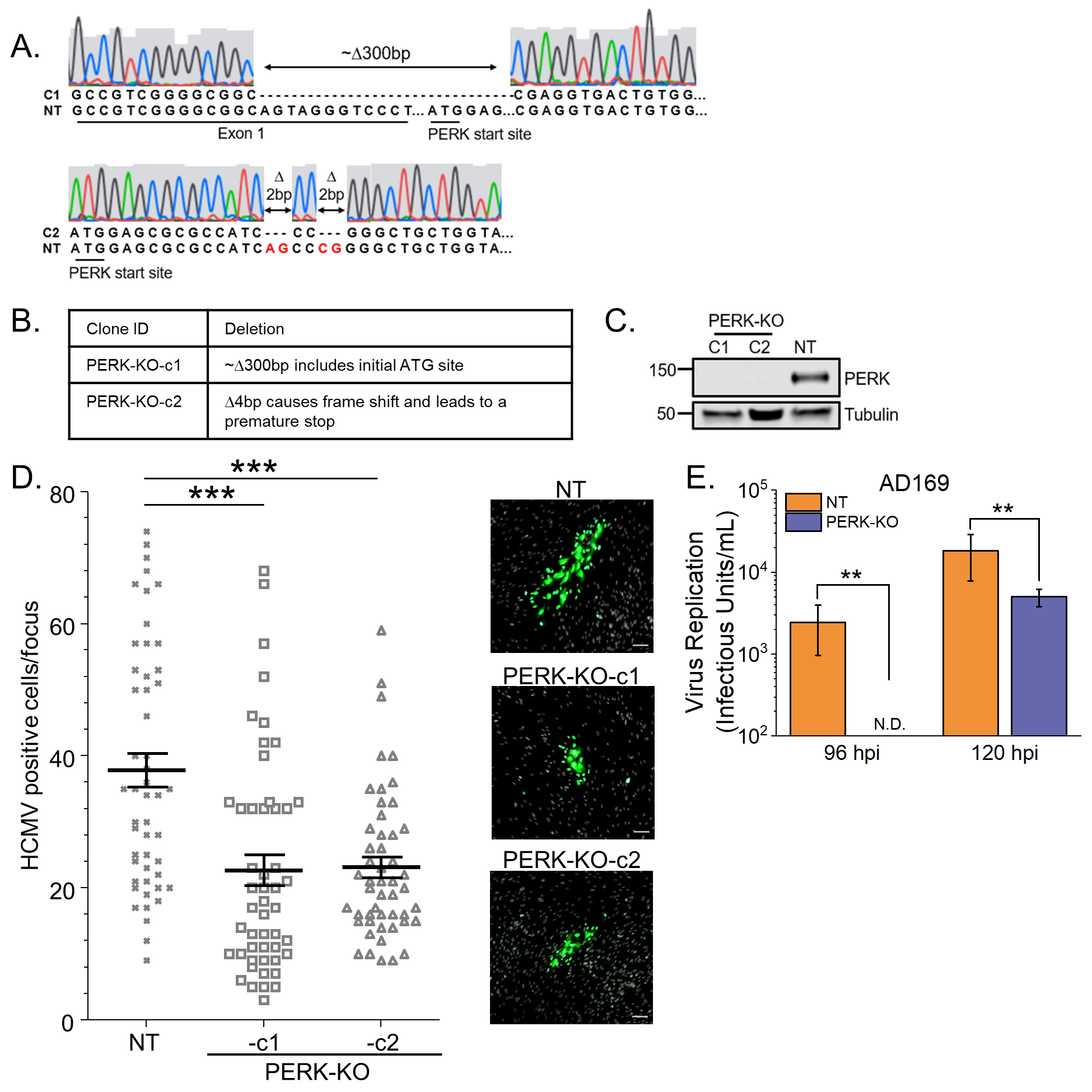

Supplement: FIG S1 [file mbio.00167-21-sf001.tif]

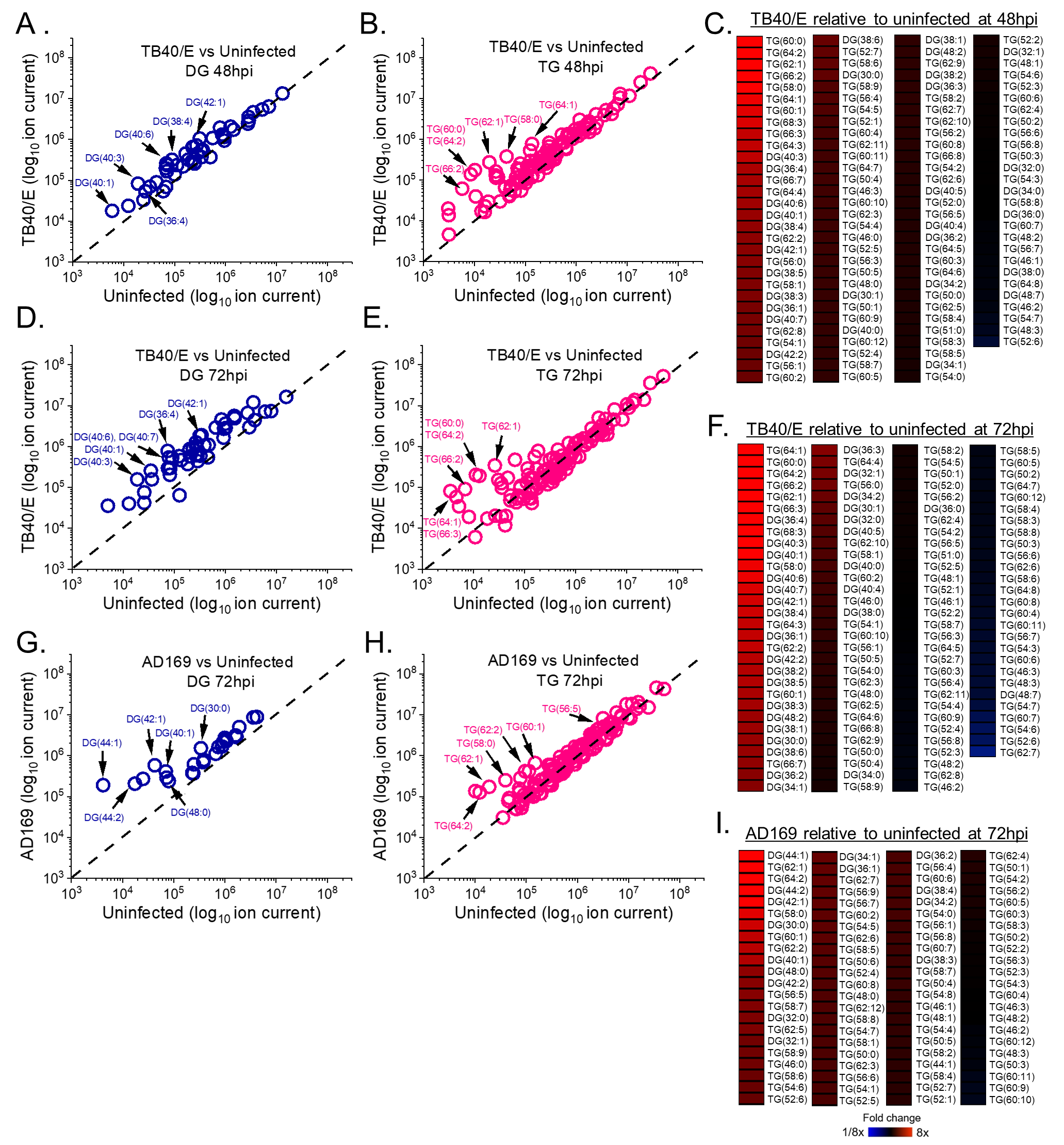

Supplement: FIG S2 [file mbio.00167-21-sf002.tif]

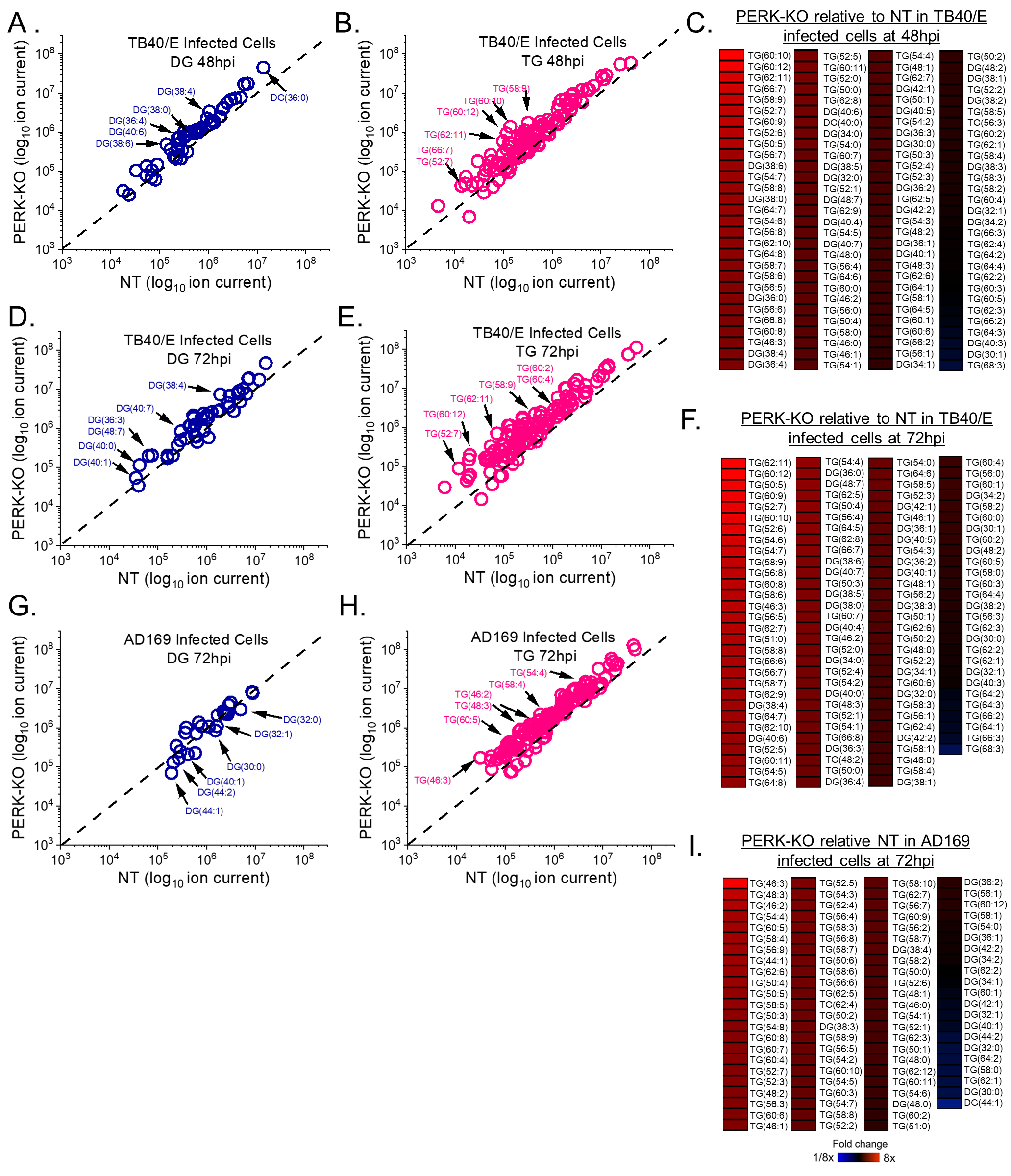

Supplement: FIG S3 [file mbio.00167-21-sf003.tif]

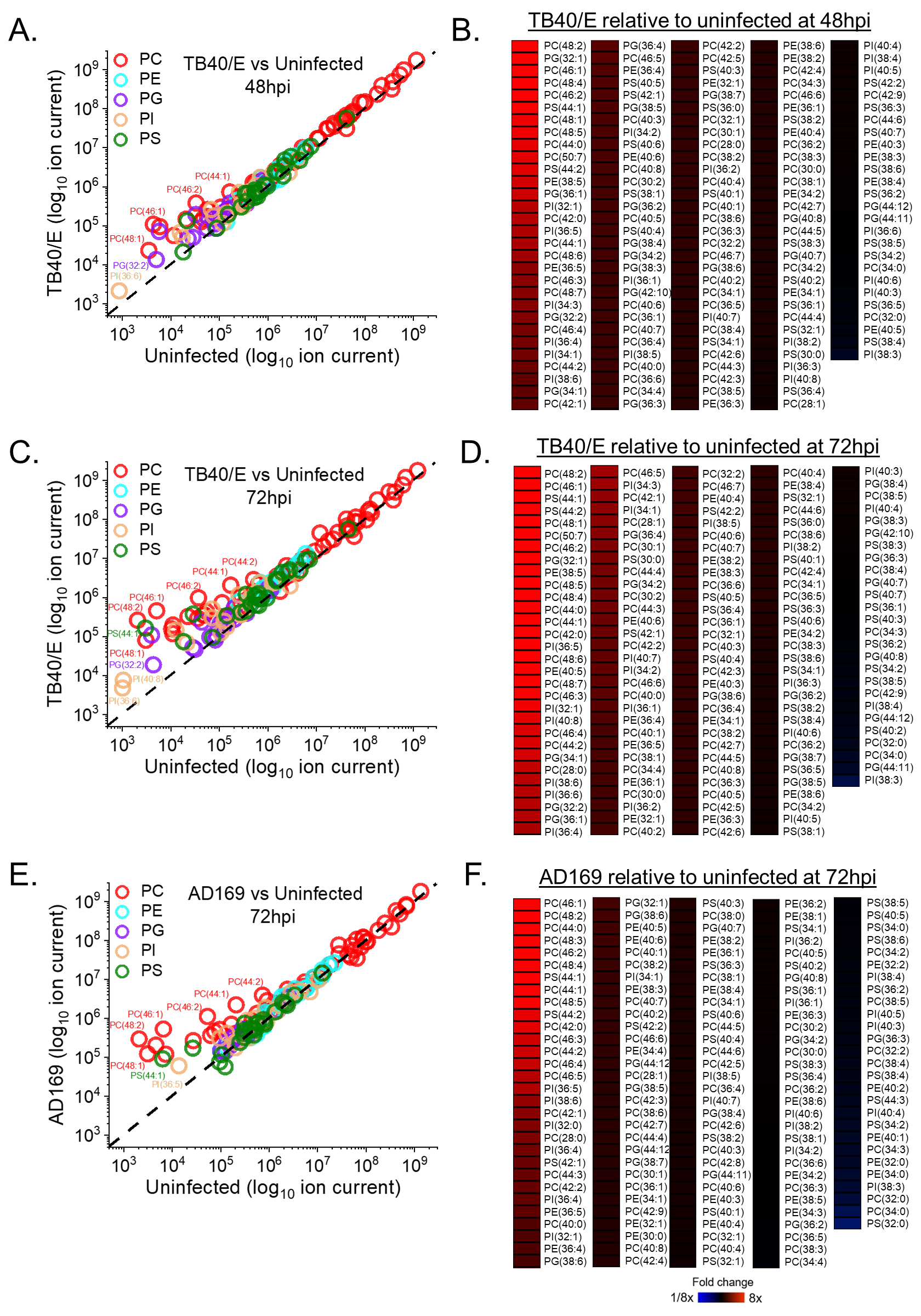

Supplement: FIG S4 [file mbio.00167-21-sf004.tif]

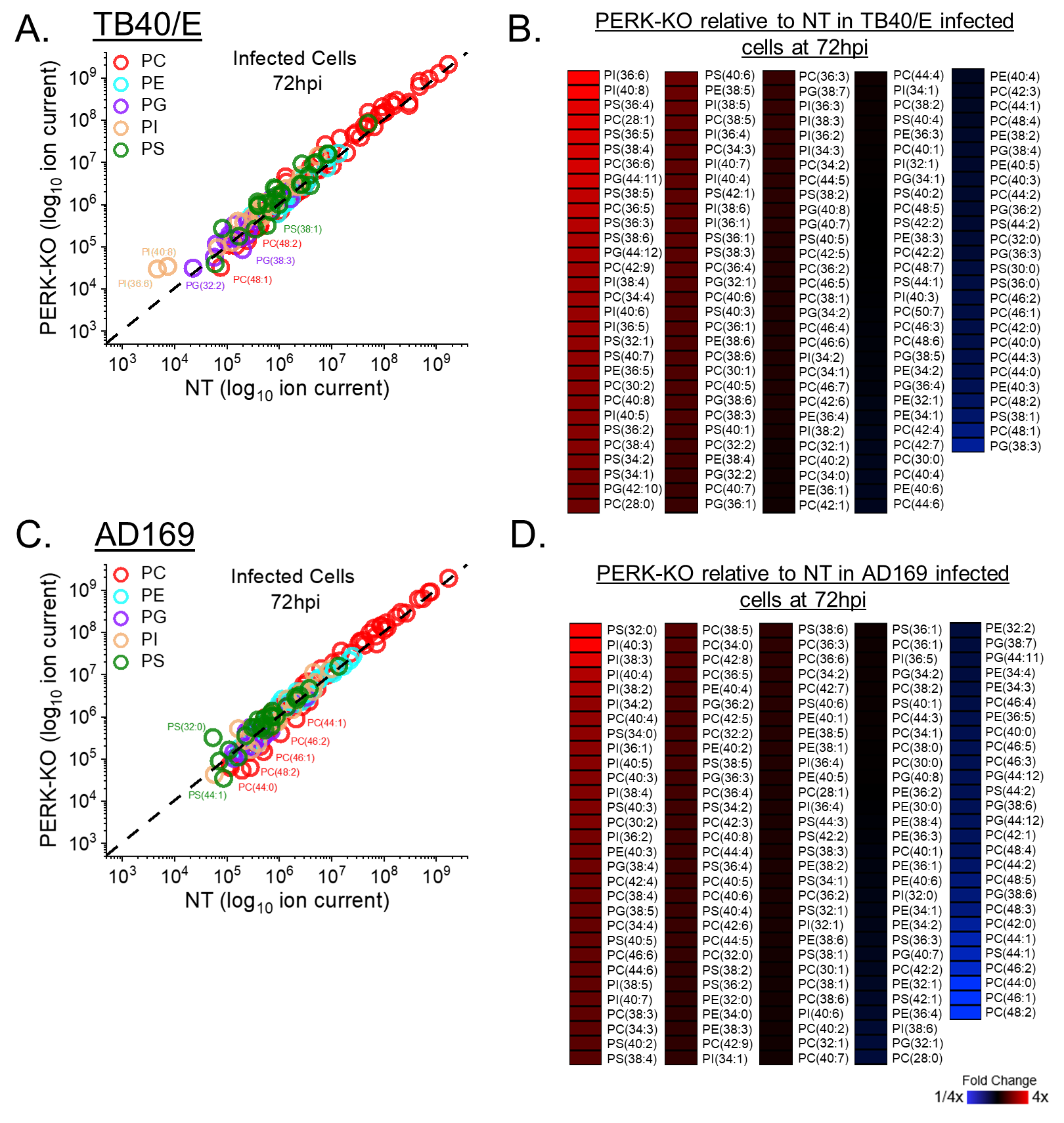

Supplement: FIG S5 [file mbio.00167-21-sf005.tif]

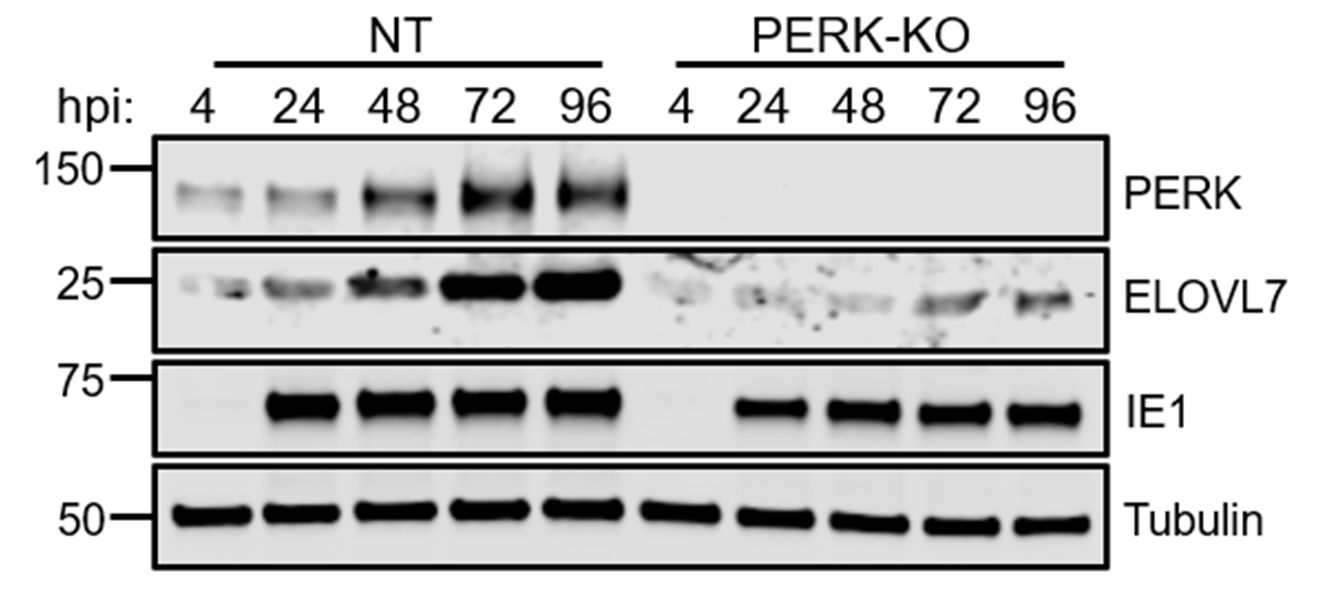

Supplement: FIG S6 [file mbio.00167-21-sf006.tif]
